# Differences in Tribological Behaviors upon Switching Fixed and Moving Materials of Tribo-pairs including Metal and Polymer

**DOI:** 10.1038/s41598-017-13262-x

**Published:** 2017-10-12

**Authors:** Aijie Xu, Pengyi Tian, Shizhu Wen, Fei Guo, Yueqiang Hu, Wenpeng Jia, Conglin Dong, Yu Tian

**Affiliations:** 1 State Key Laboratory of Tribology, Tsinghua University, Beijing, 100084 P.R. China; 2 Locomotive & Car Research Institue, China Academy of Railway Sciences, Beijing, 100081 P. R. China

## Abstract

The coefficient of friction (COF) between two materials is usually believed to be an intrinsic property of the materials themselves. In this study, metals of stainless steel (304) and brass (H62), and polymers of polypropylene (PP) and polytetrafluoroethylene (PTFE) were tested on a standard ball-on-three-plates test machine. Significantly different tribological behaviors were observed when fixed and moving materials of tribo-pairs (metal/polymer) were switched. As an example, under the same applied load and rotating speed, the COF (0.49) between a rotating PP ball and three fixed H62 plates was approximately 2.3 times higher than that between switched materials of tribo-pairs. Meanwhile, the COF between H62 and PTFE was relatively stable. The unexpected tribological behaviors were ascribed to the thermal and mechanical properties of tribo-pairs. Theoretical analysis revealed that the differences in the maximum local temperature between switching the fixed and moving materials of tribo-pairs were consistent with the differences in the tested COF. This result indicated the precise prediction of the COF of two materials is complexcity, and that thermal and mechanical properties should be properly considered in designing tribo-pairs, because these properties may significantly affect tribological performance.

## Introduction

Friction is a natural phenomenon that usually involves various physical and chemical processes^1,2^. Considerable research has been undertaken to obtain an extensive understanding and prediction of the tribological behaviors of materials so as to guide the design and application of various tribo-pairs^3–5^. Given that the two parts of a tribo-pair of the same materials can be easily welded together^6^, different materials are the more commonly selected for tribo-pairs in industrial applications.

Heat generation at the frictional interface, is an ongoing important research topic in tribology. Frictional heat can lead to the decomposition of minerals such as lubricants^7^. Therefore, the thermal effect has been widely studied in lubricantion^8^. However, the influence of thermal effect on frictional force in dry friction has yet to be fully disclosed^8–10^. Recently, Zhang *et al*. observed an increasing trends of COF and temperature of a sliding Si tip on a supported graphene surface, the trend was ascribed to the suppression of thermal lubrication and the corrugation of structures^11^. Laux *et al*. stated that adhesive friction occured between the friction of a dynamic polyetheretherketone and a static steel, resulting from high local frictional heat accumulated at the frictional interface^12^. In addition, interfacial temperature has been studied by estimating the net contact area^13^.

However, given that the mechanical properties of materials would invariably change with a variation in temperature. The frictional heating not only changes temperature of the sliding interface, but also has a complex effect on the frictional performance of materials. This effect is particularly observed in polymer materials, which may be easily changed from an elastic state to a highly viscoelastic state^14–21^. Person^14^ has developed a non-linear theory of viscoelasticity affected friction, where the ascent rate of temperature depends on the heat energy produced by local friction (related to surface roughness and sliding velocity)^15,16^. The heat diffused from friction and the nanometer interface layer has been used to explain the friction and wear of rubber tires rolling on the ground^17,18^. According to the theories of viscoelasticity and heat transfer, sliding friction at the interface is also a thermally activated process that effectively reduces barriers and increases temperature^19,20^. In addition, Babuska *et al*. revealed that polytetrafluoroethylene (PTFE) could recrystallize along the interface when interfacial temperature exceeded 127 °C, and caused the increase in COF^21^.

The COF of two different materials (such as metal and polymer) should remain constant at the same applied load and rotating speed^22,23^. But Moghadas, *et al*. found that the COF of a rotating metal (Co-27Cr-5.5Mo-0.06 C) ball friction with polymer (ultra-high molecular weight polyethylene) socket was lower than that of the switched materials of tribo-pairs^24^, because of polymer deformation without considering thermal effect. In this study, the difference in COF between the two materials of metal and polymer were observed after switching the fixed and moving parts on a test rig of an upper rotating ball rubbing on three lower fixed plates. Thermal analysis was conducted to explain the observed phenomena. The disclosed effect of switching fixed and moving materials of tribo-pairs may present important implications in improving the performance of tribo-pairs in industrial application.

## Results and Discussions

### Experimental Results of COF

**Figure 1:**
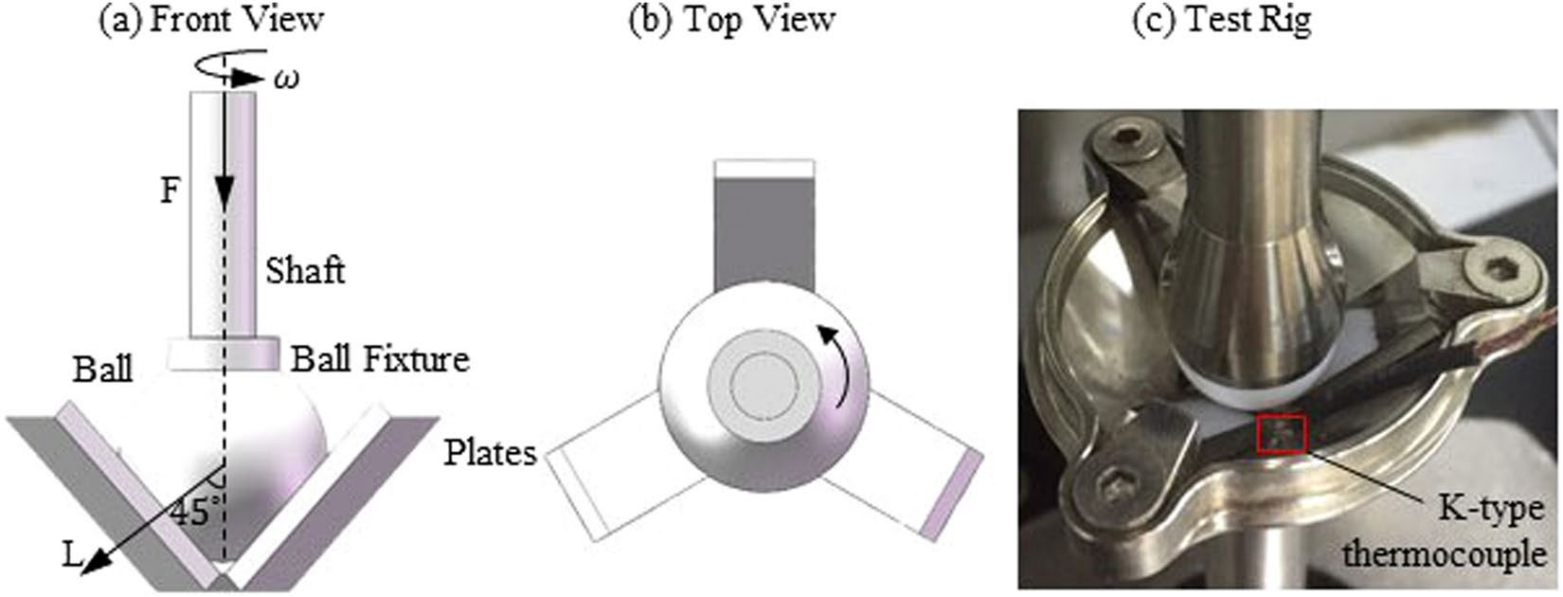
Schematic of the ball-on-three-plates friction test rig. (**a**) Front view. (**b**) Top view. (**c**) Picture of test rig.

**Figure 2:**
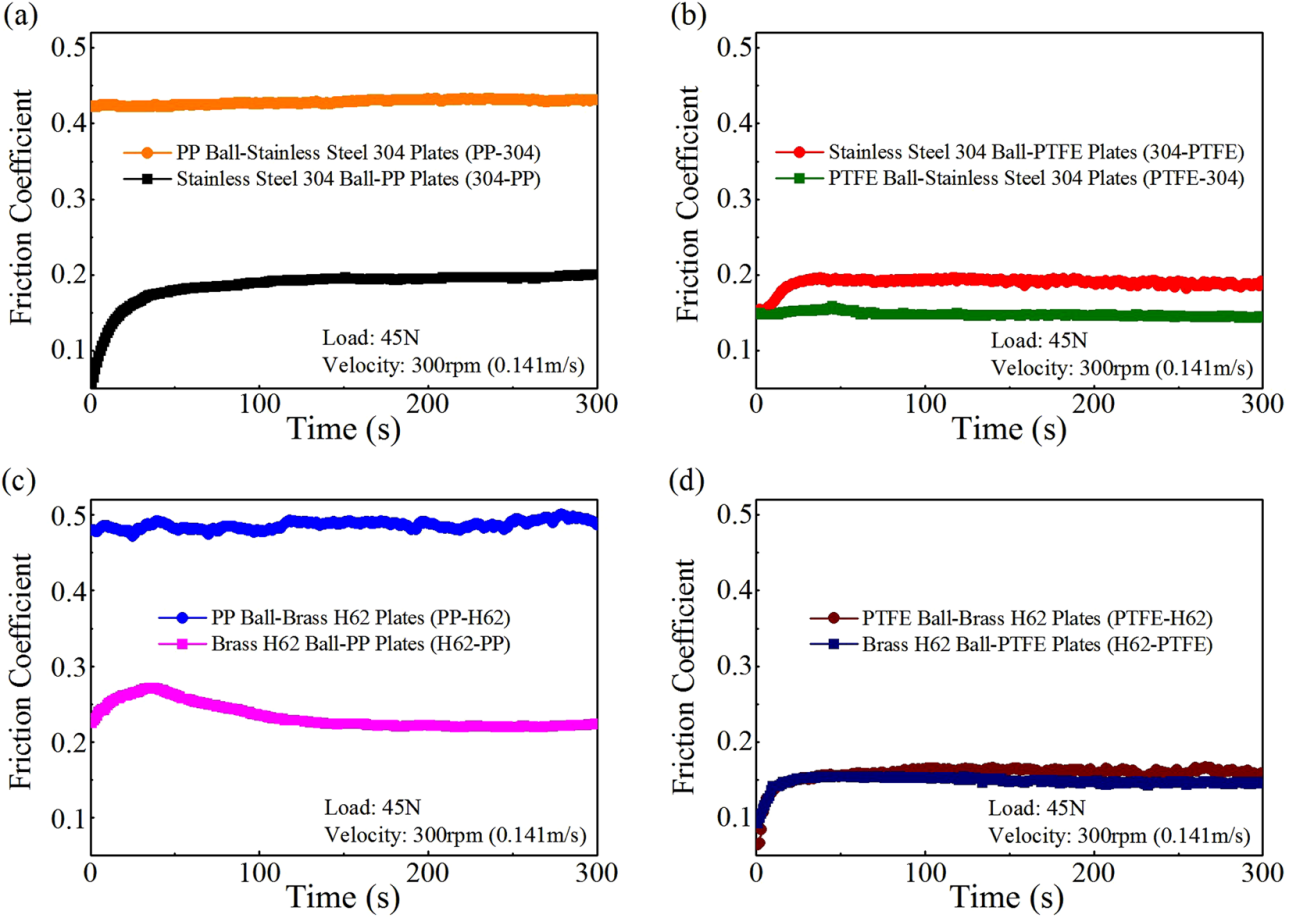
The experimental results of COF for different tribo-pairs including metal and polymer. (**a**) PP versus 304. (**b**) 304 versus PTFE. (**c**) PP versus H62. (**d**) PTFE versus H62.

A standard ball-on-three-plates module of Rheometer MCR 301 (Anton Paar Co. Ltd, Germany) was used, as shown in Fig. 1. The absolute values of COF for different tribo-pairs during rubbing within 5 min are shown in Fig. 2. The COF of PP-304 is 0.43, which is considerably higher than 0.2 of 304-PP, as shown in Fig. 2(a). The COF of 304-PTFE is a slightly higher than that of PTFE-304, and the trends of COF are opposite to the variations of other tribo-pairs, as shown in Fig. 2(b). The COF of PP-H62 exceeded that of H62-PP, as shown in Fig. 2(c). However, the difference in the COF between PTFE-H62 and H62-PTFE was minimal, as shown in Fig. 2(d).

### Theoretical Interface Temperature

**Figure 3:**
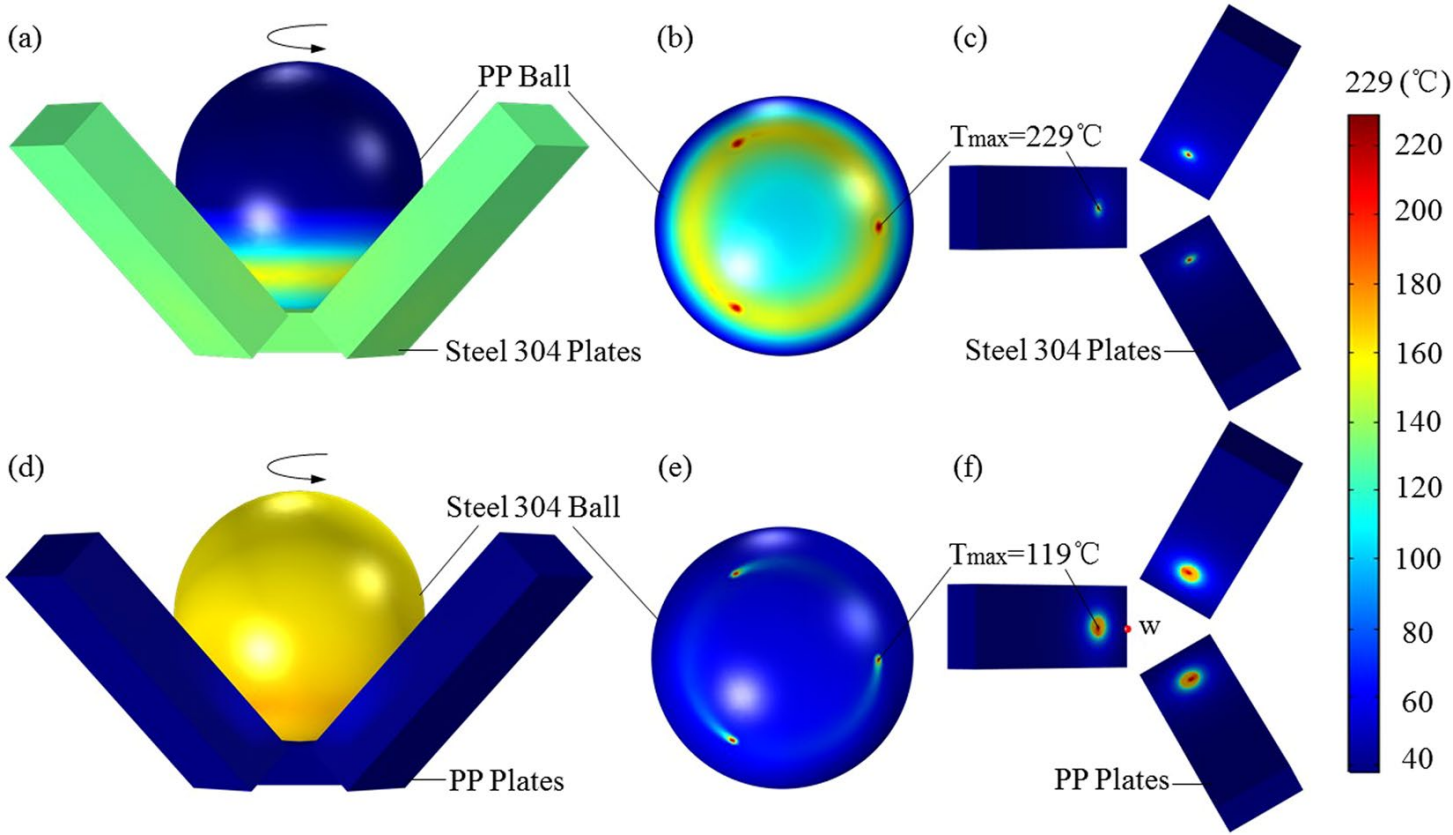
The temperature distribution of a rotating ball rubbing on thee fixed plates after 5 minutes. (**a**) Full model of PP-304. (**b**) A moving PP ball. (**c**) Three fixed 304 Plates. (**d**) Full model of 304-PP. (**e**) A moving 304 ball. (**f**) Three fixed PP Plates.

By considering that frictional power is mostly converted into heat at the sliding interface^9^, the maximum temperature (*T*_max_) of tribo-pairs at the frictional contact zone was analyzed through the solid heat transfer module of the multi-physics field in COMSOL 5.1. Typical thermo-analysis results of a rotating polymer ball sliding against three fixed metal plates in contact with PP-304, and the rotating metal ball rubbing on three static polymer plates in contact with 304-PP are shown in Fig. 3.

**Figure 4:**
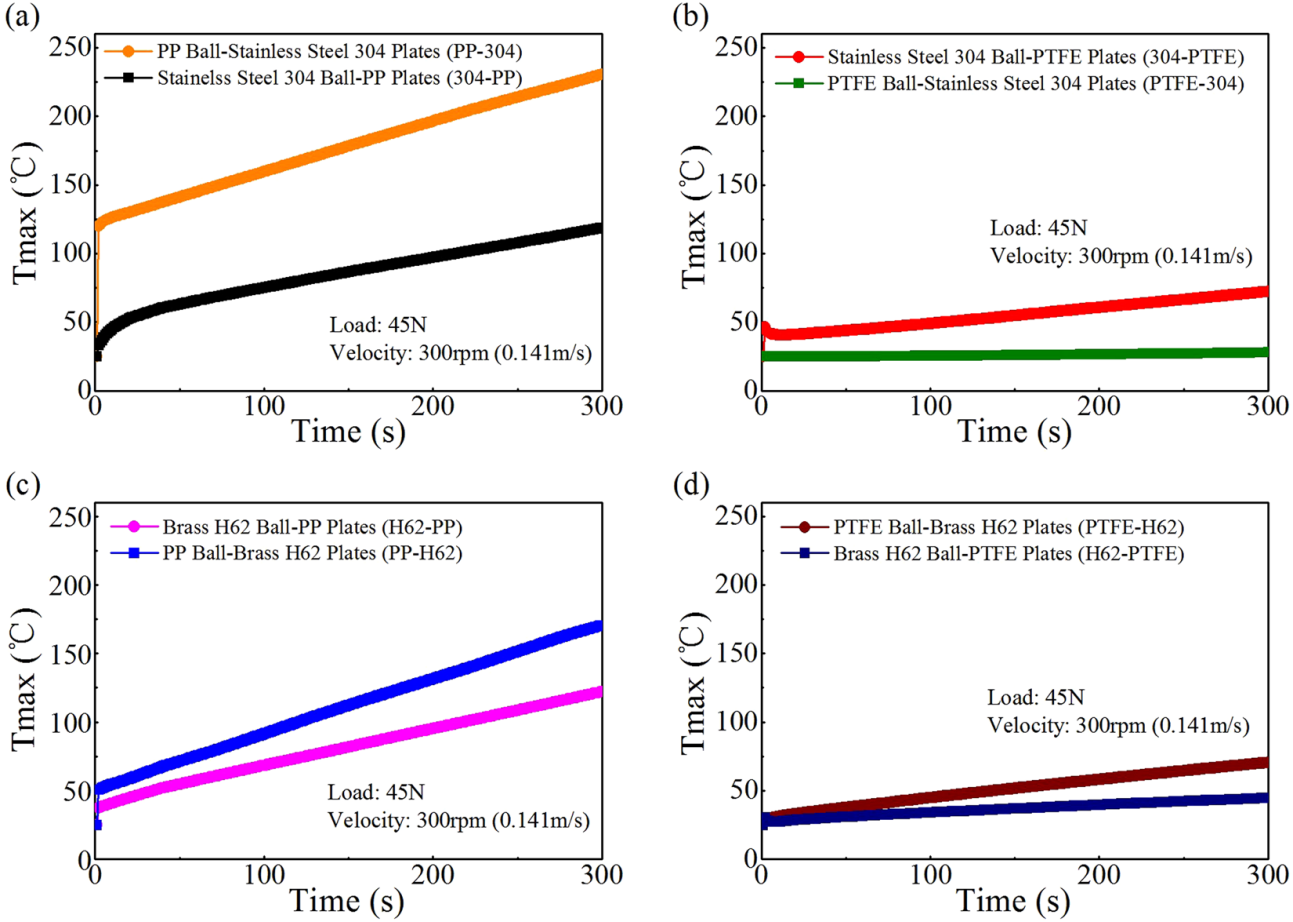
The *T*_max_ in the contact region of friction as simulated by COMSOL for different tribo-pairs. (**a**) PP versus 304. (**b**) 304 versus PTFE. (**c**) PP versus H62. (**d**) PTFE versus H62.

The *T*_max_ in the contact zone for different tribo-pairs was simulated by COMSOL for 5 min of friction, as shown in Figs 3 and 4. The results were monotonically increasing with the time going on, which were consistent with the trends of COF shown in Fig. 2. Evidently, the difference in *T*_max_ between PP-304 and 304-PP was significant, as shown in Figs 3 and 4(a). Likewise, the *T*_max_ of PP-H62 exceeded that of H62-PP, as shown in Fig. 4(c). The *T*_max_ of PTFE-H62 was slightly higher than that of H62-PTFE, as shown in Fig. 4(d). The *T*_max_ of PTFE-304 was lower than that of 304-PTFE, as shown in Fig. 4(b).

Figures 2 and 4 show that the COF on the two switching situations are consistent with the trends of increased temperature for asymmetric friction (different materials of tribo-pairs) in the contact over a small area, that is, the larger the COF is, the higher the *T*_max_ is, and vice versa. For instance, Fig. 2(a) shows that the COF of PP-304 was significantly larger than that of 304-PP. Meanwhile, Fig. 4(a) shows that the *T*_max_ (229 °C) of PP-304 was higher than that of 304-PP (119 °C), and 229 °C was evidently higher than the melting point of PP (167 °C). These results easily led to adhesive wear between the rubbing surface^25^, meanwhile, led to the increase of COF of PP-304.

For 304-PP and H62-PP, the elasticity modulus of PP (1 GPa) is lower than those of Steel 304 (272 GPa) and brass H62 (94 GPa), as shown in Table S1 of the Supporting Information. The COF gradually increased at the initial running-in stage when PP was elastically deformed, and then the COF of 304-PP and H62-PP remained stable until the plastic deformation of PP occurred. The above explanation was also applicable to 304-PTFE and H62-PTFE (0.62 GPa), as shown in Fig. 2.

**Figure 5:**
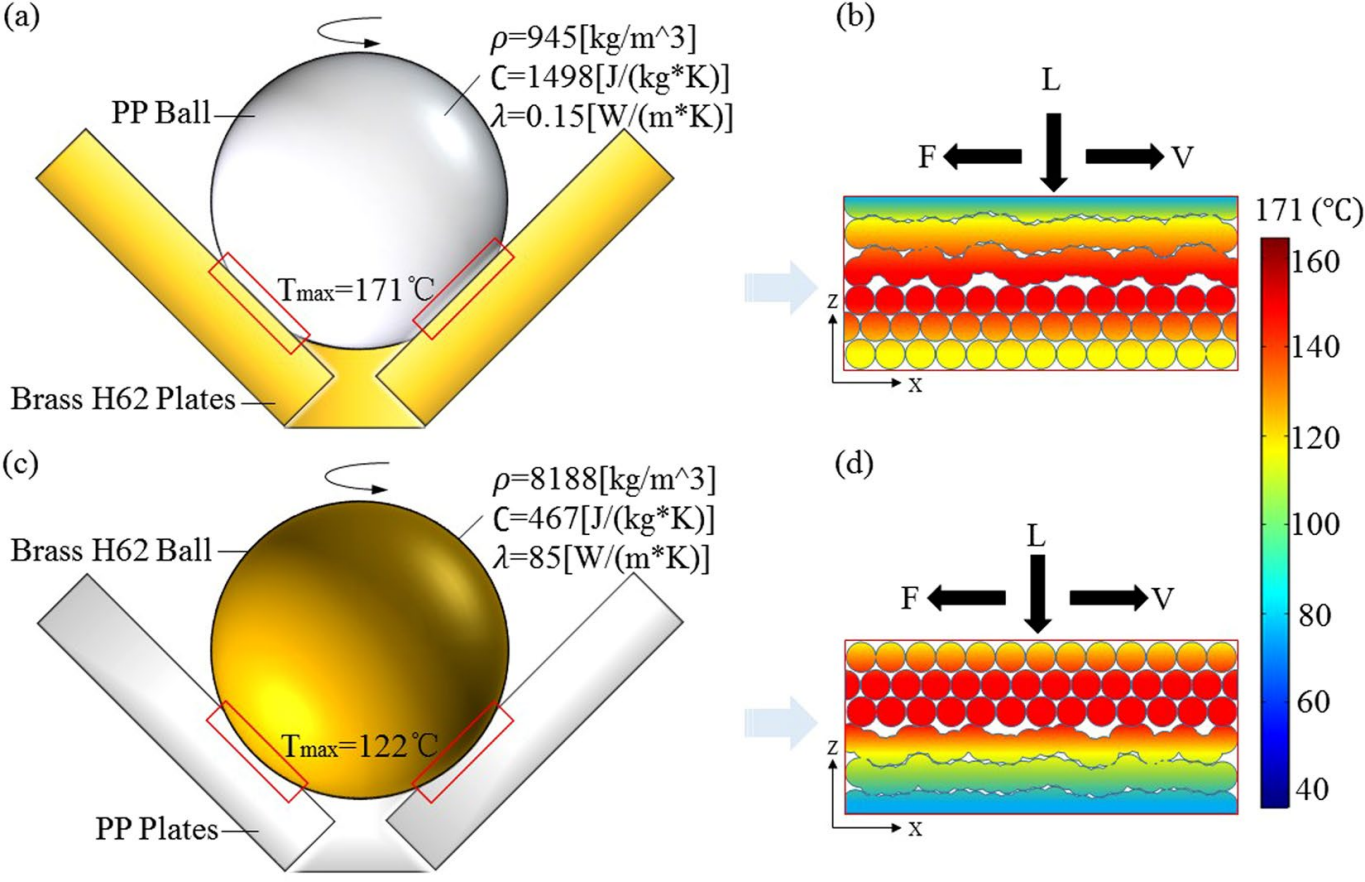
Schematic: the distribution of heat flux across the frictional contact interface of PP-H62 and H62-PP after 5 min friction. (**a**) A dynamic PP ball rubbing on three static brass H62 plates (PP-H62). (**b**) Thermal diffusivity of dynamic PP ball was relatively low in the vicinity of the fricitional contact region, in which more frictional heat was more accumulated near the contact interface (as shown in Fig. 3(b)), and the *T*_max_ of PP-H62 was 171 °C. (**c**) A dynamic brass H62 ball rubbing on three static polymer PP plates (H62-PP). (**d**) Thermal diffusivity of dynamic brass H62 ball was relatively fast in the vicinity of the frictional contact region (as shown in Fig. 3(e)), and the *T*_max_ of H62-PP was 122 °C.

**Figure 6:**
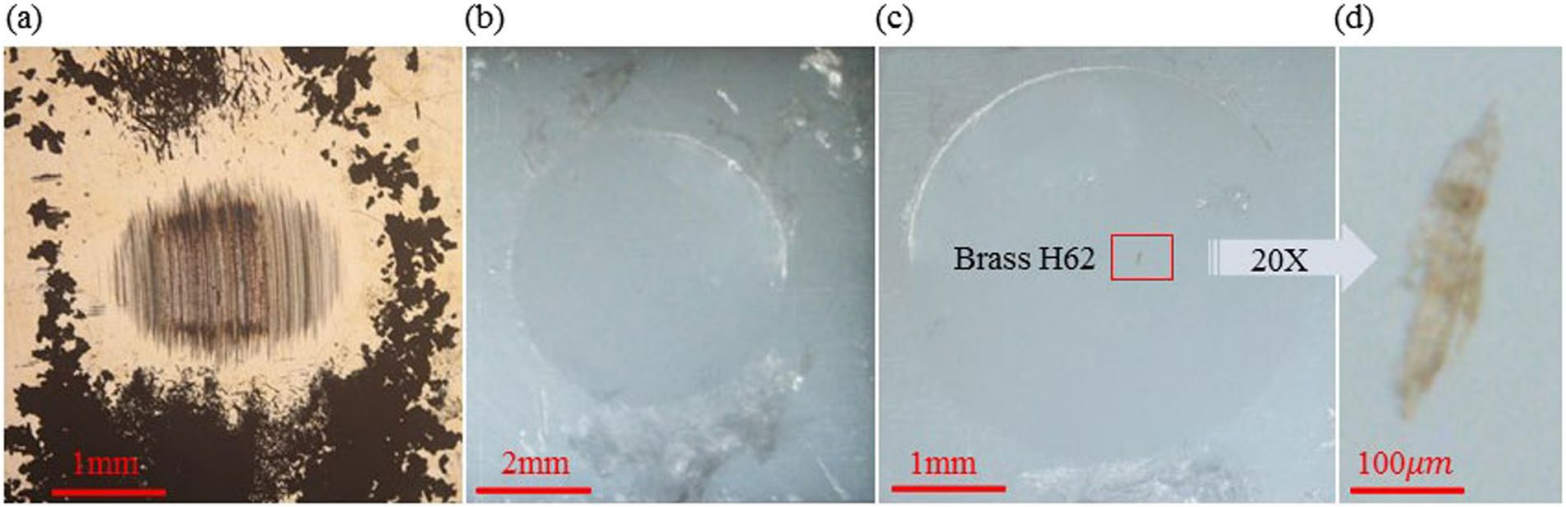
Image in wear scars and wear particles under a microscope. (**a**) Wear particles of PP-H62 that led to significant increase of the COF. (**b**) Wear debris of PTFE for 304-PTFE. (**c**) Minimal brass H62 glued onto the PTFE plate. (**d**) Magnified image of minimal brass H62.

Figure 2(c) shows that the COF of PP-H62 is markedly larger than that of H62-PP. Figure 4(c) shows that the *T*_max_ levels of PP-H62 and H62-PP are 171 °C and 122 °C, respectively. The temperature distributions of PP-H62 and H62-PP are plotted in Fig. 5. Given that the thermal conductivity of brass H62 (85 [W/(m∙K)]) is 567 times higher than that of polymer PP (0.15 [W/(m∙K)]) material and 5 times higher than that of steel 304 (17.2 [W/(m∙K)]), most of the thermal energy was diffused into the surrounding enviroment from brass H62 during friction, and then led to a relatively lower rate of temperature rise in PP-H62, compared with PP-304. Some black wear particles (Fig. 6(a)) were found in the friction of PP-H62, these particles may lead to abrasive wear, and increase the COF of PP-H62.

Figures 2(b) and 4(b) show that both the COF and temperature increment of PTFE-304 were lower than those of 304-PTFE. For the metal-to-polymer tribo-pairs, the polymer materials were easily strained during friction. The internal heat sources were connected with plastic strains and accompanied by heat energy dissipation, which resulted in a temperature increase^26^. Self-lubricating polymer materials and polymer transfer films acted as lubricants during the frictional process of 304-PTFE, as shown in Fig. 6(b), and led to a relatively lower COF than those of tribo-pairs, such as metal-to-metal pairs. For example, fluoride ions were observed on the metal surface with XPS when PTFE was rubbed against stainless steel or nickel^27^. Tanaka *et al*.^28^ suggested that a transfer film destroyed the banded structure of PTFE owing to its low activation energy (29 kJ/mol) and slippage between the crystalline slices. On the basis of electron microscopy and differential thermal analysis, Kar and Bahadur^29,30^ stated that the slippage of crystalline slices interspersed with amorphous materials contributed to inter-lamellar shear.

The creep properties of polymers with low heat conductivity strongly depend on temperature. Moreover, heat generated in friction often resulted from the deformation and removal of materials, including plastic deformation, adhesion hysteresis, dispersion, and viscous flow^31^. Figures 4(d) and 5(d) show that the COF and temperature increase of H62-PTFE and PTFE-H62 were in a good agreement. Moreover, the wear particles of brass H62 can easily adhere to the PTFE surface at the contact area of friction, as shown in Fig. 6(c) and (d). The metal-to-polymer contact of H62 and PTFE can be easily inter-transferred to their interface^32,33^. Brass H62 particles were embedded in PTFE and easily caused abrasive wear, and polymers of low cohesive energy density transfered to the materials of higher cohesive energy density^30^. Therefore, regardless of the moving/fixed properties of PTFE/brass H62, the COF in the two situations were highly similar.

## Conclusions

Significant differences in COF and the *T*_max_ are observed by switching the fixed and moving metal/polymer tribo-pairs of a rotating ball sliding on three stable plates for asymmetric friction. The different tribological behaviors are ascribed to the thermal and mechanical properties of materials. Frictional heating can be a major factor affecting the COF, particularly, for frictional materials with low melting point and high thermal conductivity. This effect should be considered in designing fixed and moving tribo-pairs, and also implies the complexity of precisely predicting the absolute values of COF between two materials.

## Methods

### Materials and Experimental Conditions

In this study, a metal or polymer ball with a diameter *D* = 12.7mm was rotated and rubbed on three lower fixed plates with a dimension of 15 mm × 6 mm × 3 mm. Metals of stainless steel 304 and brass H62, and polymers of PP and PTFE were used. The metal and polymer balls were manufactured by using materials purchased from Steel Ball Co. Ltd. Shanghai Ningxing and Steel Ball Co., Ltd. Zhejiang Shangyu Xinxin, according to GB308-2002 standards. The plates were laser cut, engraved, and then polished on a Tegra System grinding and polishing Machine (Struers Tegramin-25, Denmark). The surface roughness and mechanical properties of the specimens are shown in Table S1 of the Supporting Information.

For a reasonable comparison of experimental results, the normal loads were all set to *F* = 45 N. The upper ball was rotated at $n$ = 300 rpm, which corresponded to a sliding velocity of *u* = 0.141 m/s. The duration of each test was *T* = 5 min (300 s), and 42.3 m in sliding distance. Prior to conducting the experiments, the metal specimens were cleaned in an ultrasonic bath with acetone at 80 °C for 15 min and then with deionized water at 30 °C for 5 min. Polymer specimens were cleaned at room temperature for 5 min with deionized water. All specimens were dried with nitrogen. All of the experiments were repeated a minimum of three times.

### Thermal Analytical Theory

The geometric configurations of the full COMSOL model were established, according to the actual sizes, as sown in Fig. S3 of the supporting information. Therefore, the heat dissipation of the model mainly originated from heat conduction among the ball, the ball fixture, and the three plates, the chamber, and the surrounding air. The wear scars of the balls and plates after different sliding times were measured by optical microscopy, and then arbitray fitted by logarithmic function of time *t*. The nominal contact radius changed over time is described as:

1 $${r}_{nom}(t)={r}_{hertz}+b\cdot \,\mathrm{ln}(n\cdot t)(t\le 300)$$

where *t* is the test time (300 s), ${r}_{{hertz}}$ is the Hertz contact radius at the static contact state, *n* is the rotating speed, and *b* is a variable related to ${r}_{{nom}}(t)$. Given that the net contact area was only a small part of the nominal contact area at the interface, frictional heating was only generated at the net contact area in the model. Thus, for simplicity, a scale factor *k* (<1) is introduced to set the net contact area ${A}_{{net}}(t)$, as shown in Eq. (2),

2 $${A}_{net}(t)=k{A}_{nom}(t)=k\pi {r}_{nom}{(t)}^{2}$$

and the net contact radius is

3 $${r}_{net}(t)=\sqrt{k}\cdot {r}_{nom}(t)$$

The stress distribution in the net contact area, based on the correction formula of Hertz contact theory is described as

4 $$p(x,y,z)={p}_{\max }(t){(1-\frac{{(x-x\text{'})}^{2}+{(y-y\text{'})}^{2}+{(z-z\text{'})}^{2}}{{r}_{net}{(t)}^{2}})}^{1/2}$$

where *x*′, *y*′ and *z*′ respectively indicate the three components of the distances in three orthogonal directions, from the center of the rotating ball (0, 0, 0). The maximum Hertz contact stress ${p}_{{\max }}(t)$ is described as

5 $${p}_{\max }(t)=\frac{3}{2}\frac{L}{{A}_{net}(t)}$$

where *L* is the normal force at three contact points of the ball and each plate, according to the geometrical relationship between *L* and *F*, *L* = *3F*/3 cos45° = 45 $\sqrt{2}$ N, as shown in Fig. 1.

Friction work was assumed to be fully transformed into heat in the model. The frictional heat power per unit area on contact interface was described as

6 $${q}_{heat}(x,y,z,t)=vcof(t)p(x,y,z,t)/3=\frac{2\pi {R}_{rot}ncof(t)p(x,y,z,t)}{3\times 60}$$

where *cof* (*t*) is the COF, *n*(*r/min*) is the rotating speed, and ${R}_{rot}$ = 4.5 mm is the gyration radius of the rotating ball friction with the three fixed plates. One-third of the total friction power was consumed at each contact point. Heat transfer is described as:;

7 $$\rho C\frac{\partial T}{\partial t}+\rho Cu\cdot \nabla T+\nabla \cdot (-\lambda \nabla T)=Q$$

where *ρ* is the material density, *C* is the heat capacity of material, *λ* is the thermal conductivity, *T* is the temperature, *Q* is the thermal power per unit volume, and *u* is the velocity of the rotating ball. For the upper ball and the ball fixture, ${{u}}_{{x}}=-{y}2\pi n{/}60$, ${{u}}_{{y}}={x}2\pi n{/}60$; for other parts, *u* = 0^34^.

### Measurment of Experimental Temperature

Given the difficulty in directly measuring temperature at the frictional contact region, we used an indirect measurement method in this study. An unshielded K-type thermocouple (−200 °C to 1372 °C) made by the Shenzhen Everbest Machinery Industry Company was fixed between one of three plates and the wall of test chamber, as shown in Fig. 1. Point “*w*” (about 3/$\sqrt{2}$ mm from the frictional contact point at the *Y* direction) was measured in a continuous rotating test within 5 min under the conditions of 45 N and 300 rpm, as shown in Fig. 3(f). The relevant calibration parameters of different tribo-pairs are shown in Table 1.

**Table 1:** Adjusted parameters of different tribo-pairs.

Tribo-pairs (abbr.)	*r*_*hertz*_[mm]	*b* [mm]	*k*	Temperature Difference (°C)
stainless steel 304 ball-PP plates (304-PP)	0.62	0.004	0.1	0.4
stainless steel 304 ball-PTFE plates (304-PTFE)	0.74	0.21	0.1	0.77
brass H62 ball-PTFE plates (H62-PP)	0.62	0.008	0.1	0.6
brass H62 ball-PP plates (H62-PTFE)	0.75	0.11	0.1	0.14

**Figure 7:**
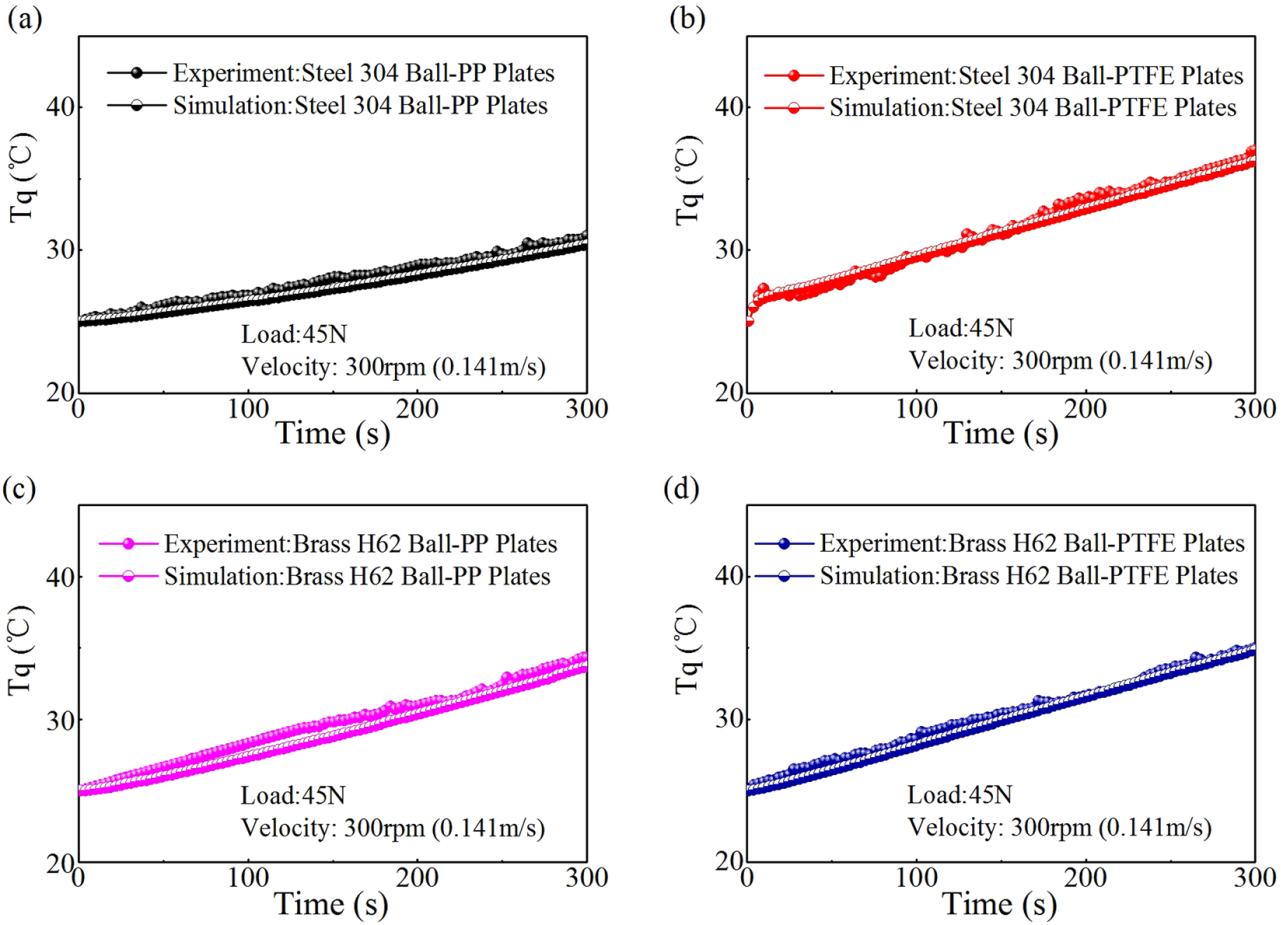
Measured temperature and simulated temperature (COMSOL) at the same point “*w*”. (**a**) PP versus 304. (**b**) 304 versus PTFE. (**c**) PP versus H62. (**d**) PTFE versus H62.

The measured temperature at point “*w*” was used to adjust the relative parameters “*b*” (Eq. (1)) and “*k*” (Eq. (2)) in the COMSOL model^8,35^, as shown in the above Table 1. This approach was used to make the simulation results nearly coincide with the temperature increase measured by the K-type thermocouple, as shown in Fig. 7. In Fig. 7 and Table 1, the gap of the *T*_max_ between the simulations and measurements at poin “*w*” did not exceed 0.8 °C within 5 min. Therefore, the calculation method was reasonable.

## Electronic supplementary material

Supplementary Information
