# Supplementary material for: Differences in Tribological Behaviors upon Switching Fixed and Moving Materials of Tribo-pairs including Metal and Polymer
Source: Sci Rep. 2017 Oct 12;7:13041. doi: 10.1038/s41598-017-13262-x (PMC5638825; doi:10.1038/s41598-017-13262-x)
Supplement: Supplementary file 1 — Supplementary Information [file 41598_2017_13262_MOESM1_ESM.doc]

**Differences in Tribological Behaviors upon Switching Fixed and Moving Materials of Tribo-pairs including Metal and Polymer**

Aijie Xu^1^, Pengyi Tian^1,2^, Shizhu Wen^1^, Fei Guo^1^, Yueqiang Hu^1^, Wenpeng Jia^1^, Conglin Dong^1^, Yu Tian^1,*^

^1^State Key Laboratory of Tribology, Tsinghua University, Beijing 100084, P.R.China.

^2^Locomotive & Car Research Institue, China Academy of Railway Sciences, Beijing 100081, P. R. China

*Corresponding.tianyu@mail.tsinghua.edu.cn

**Supplementary Information**

**Ball-on-disk Test (UMT).** The ball-on-disk test was conducted on the Universal Mechanical Tester (UMT), Bruker Corporation, Campbell, CA∙USA, with a polymer/metal ball (∅12.7mm) and a metal/polymer disk (∅24×8mm). The experimental results were qualitatively consistent with the ball-on-three-plates experiment in this manuscript. Under the conditions of applied load of 20N and sliding speed of 7mm/s, the trends of coefficient of friction (COF) were nearly consistent with the ones of ball-on three plates in this manuscript, although the absolute values of the COF were not completely the same as this manuscript. Meanwhile, ref. 24 has disclosed the same phenomenon. The results of the mean COF of the ball-on-disk on UMT, as shown in Fig. S1.


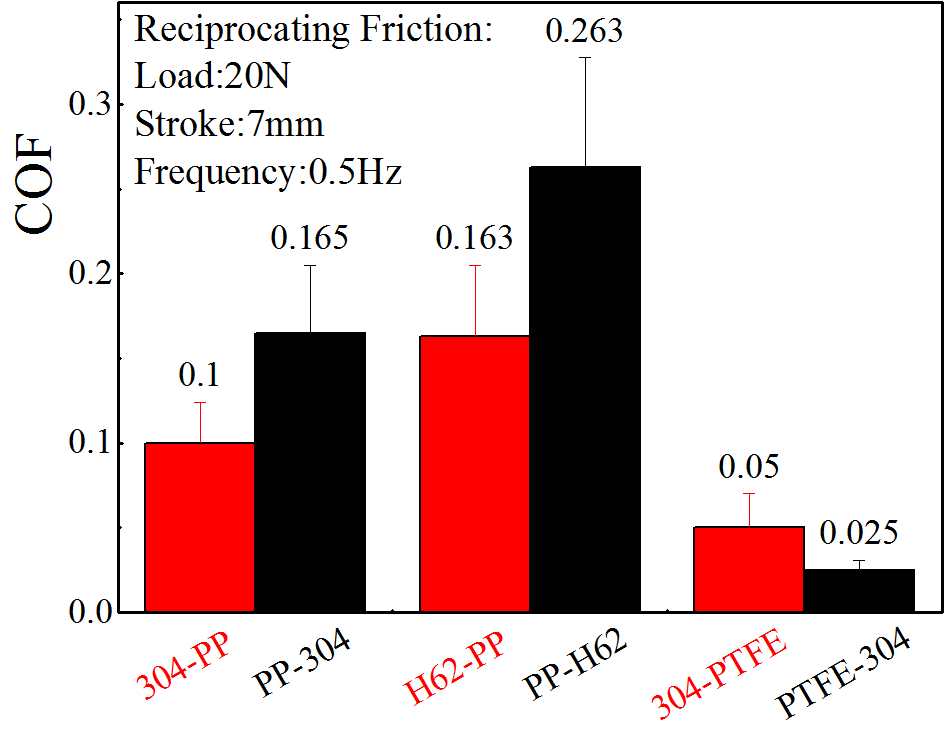


Figure S1. The COF of ball-on-disk on UMT

**Four-ball Test (FBT)**. The four-ball test on the Four-ball Test Machine produced by Xiamen Tenkey Automation Co. Ltd, with the diameters of the four balls are 12.7mm, as shown in Fig. S2.


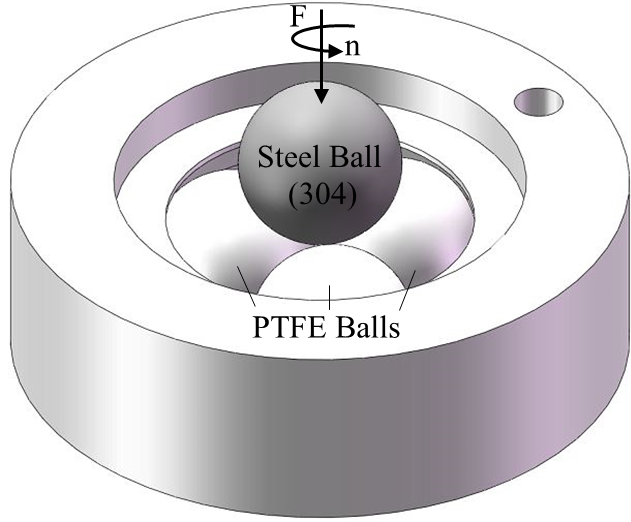


Figure S2. Schematic diagram of Four-Ball Test

The trends of COF in the experimental results were nearly consistent with the ones of ball-on three-plates in this manuscript, although the absolute values of COF were not completely the same as this manuscript. Even though for different conditions of applied load (100N) and rotating speed (300rpm) within 300 seconds, compared with the loading conditions of the manuscript. Meanwhile, ref. 24 has disclosed the same phenomenon. The results of the mean COF of the four-ball tested on the Four-Ball Test Machine, as shown in the following Fig. S3.


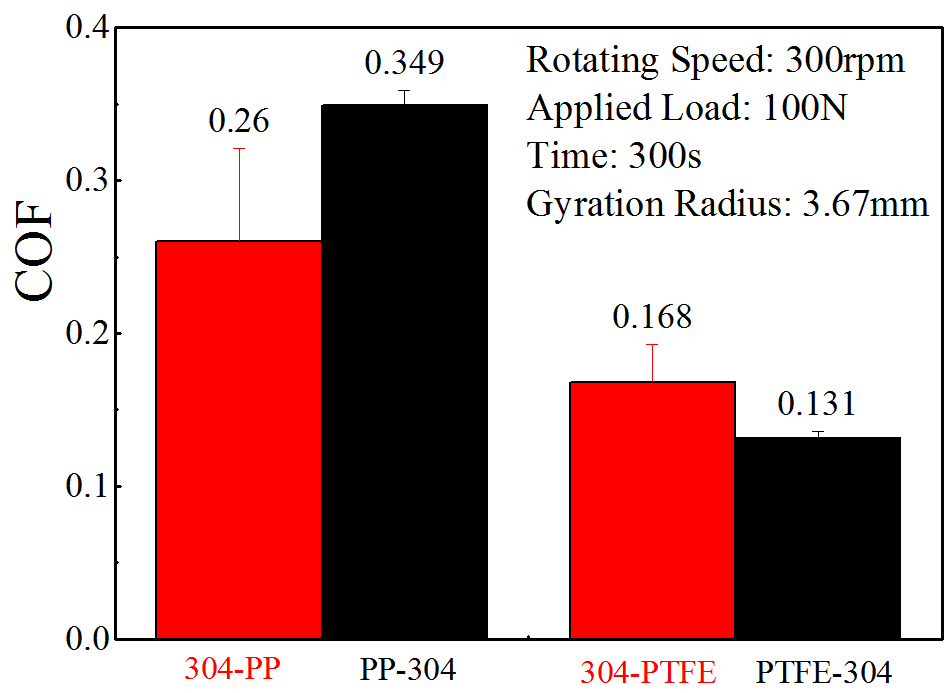


Figure S3. The COF of the FBT

**Cooling Test.** To quickly and accurately obtain cooling temperature during rotating friction, liquid nitrogen gas was introduced into closed chamber with 4°C/ min, keeping the temperature at -80°C. The liquid nitrogen gas could help taking away the heat and effectively reduce the absolute values of COF, as shown in Fig. S4.


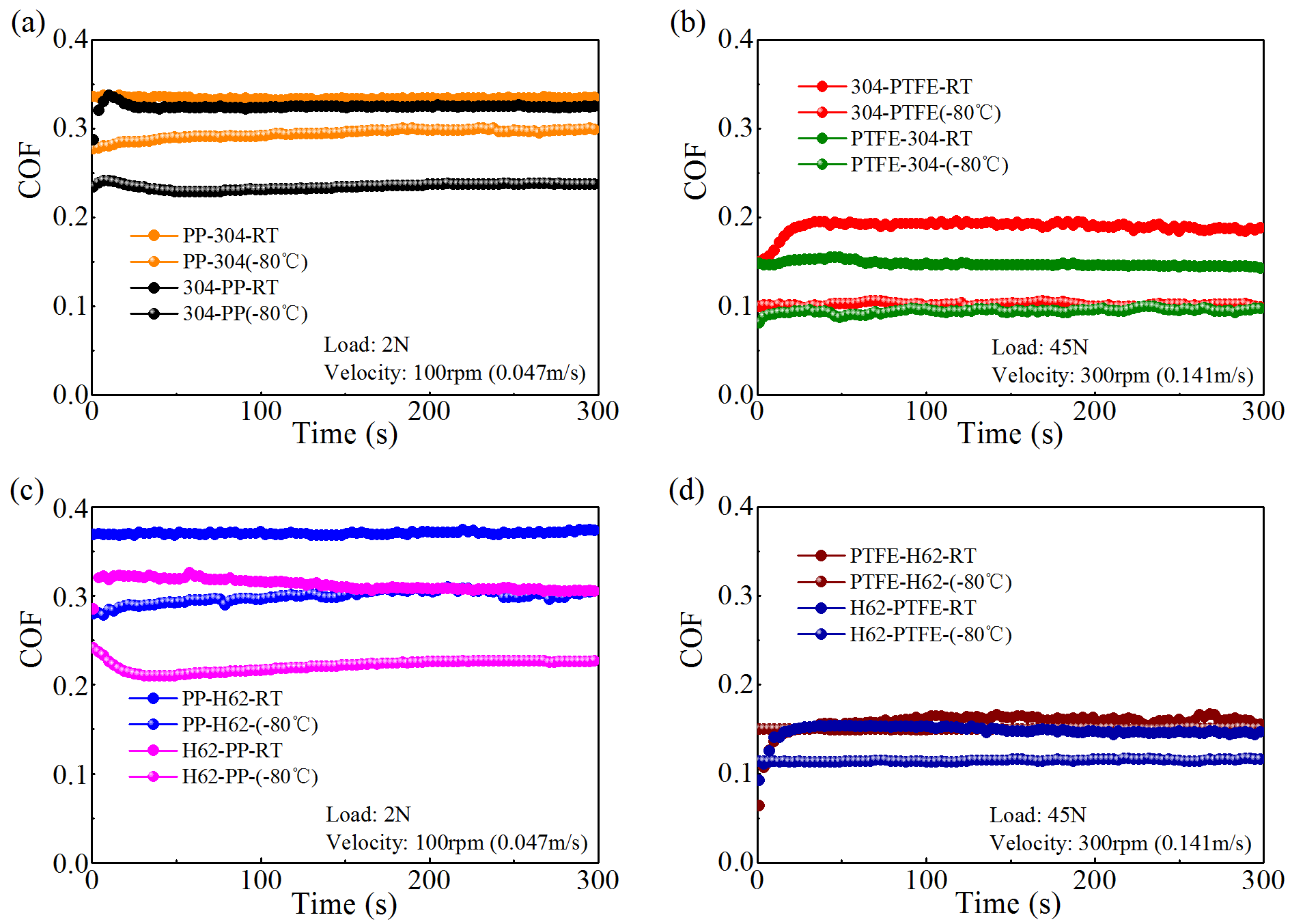


Figure S4. The absolute values of COF for different tribo-pairs including metal and polymer on room temperature (25°C) and cooling temperature (-80°C)

For the asymmetric materials of tribo-pairs (also called asymmetric friction) in the cooling test, the trends of COF at -80°C was nearly in accordance with the tests at room temperature, although the absolute values of COF at -80°C were evidently lower than the ones of room temperature, i.e. the absolute values of COF PP-304, PP-H62, PTFE-H62 and 304-PTFE were respectively higher than 304-PP, H62-PP, H62-PTFE and PTFE-304, as shown in Fig. S4. The reason is that more heat energy diffused into the cooling surrounding environment, the less thermal energy accumulated at the contact zone of friction, ultimately, resulting into a much lower absolute values of COF at -80°C, compared with room temperature. The results further proved that the frictional heating is mainly related with the frictional materials themselves.

On account of polymer PP will lead to severe rubbing against metal (304 and H62) under 45N and 300 rpm at cooling temperature (-80°C), in order to protect the frictional testing instrument, we only measured the friction coefficients of PP-304, PP-H62, 304-PP and H62-PP under the conditions of 2N and 100rpm.

**COMSOL Model**. The COMSOL model (including a ball, three plates and the surrounding clamps) has established, according to the actual sizes, as shown in the Fig. S5. In order to clearly show the results of thermal analysis, we have hidden other parts of the whole model in this manuscript, except a ball and three plates.


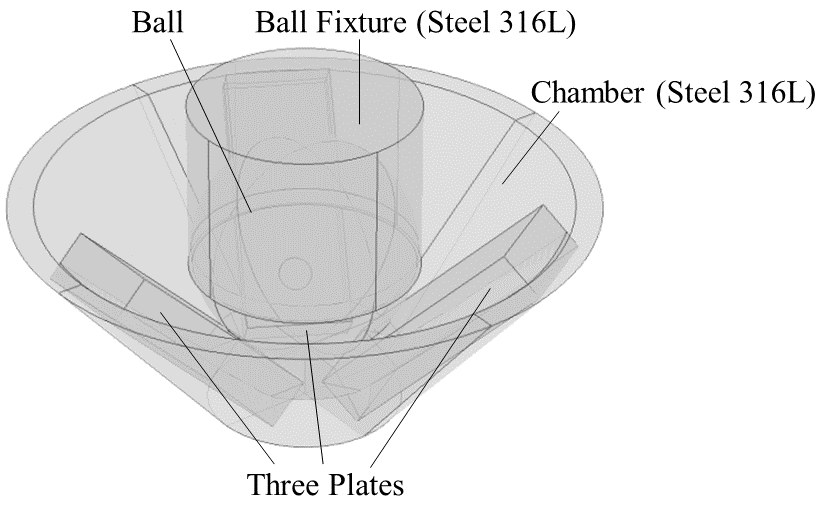


Figure S5. The geometric configurations of full COMSOL model

**Parameters of COMSOL Model.** The specific heat capacity (C), thermal conductivity (λ) and elastic modulus (E) of PP, PTFE, Steel 304 and Brass H62 measured by Quantum Design PPMS-9 and Hysitron TI 950 TriboIndenter, other specific parameters of this COMSOL model, as shown in Table S1and Table S2.

| Symbol | Description | Value | | | | | |
| --- | --- | --- | --- | --- | --- | --- | --- |
|  |  | Steel  (304) | Brass  (H62) | Polymer  (PP) | Polymer  (PTFE) | Steel  (316L) | Air |
| $\rho$ | Density (kg/m^3^) | 7726 | 8188 | 945 | 2143 | 8000 | 1.1697 |
| *C* | Specific Heat Capacity (J/kg∙K) | 591.3 | 467 | 1498 | 467.5 | 500 | 1100 |
| *λ* | Thermal Conductivity (W/(m∙K)) | 17.2 | 85 | 0.15 | 0.3 | 16.3 | 0.026 |
| *E* | Elastic Modulus (GPa) | 272.15 | 93.64 | 1.08 | 0.62 | 193 | - |
| *μ* | Poisson Ratio | 0.3 | 0.3 | 0.4 | 0.4 | 0.3 | - |
| *ε* | Infrared Emissivity | 0.05 | 0.04 | 0.91 | 0.9 | 0.05 | - |
| *H* | Hardness [HV] | 194 | 172 | 7.24 | 2.7 | - | - |
| *Ra* | Surface Roughness (nm) | 63/56 | 89/75 | 48/40 | 83/80 | - | - |
| *T_air* | Ambient Temperature ( °C) | 25 | | | | | |
| $R$ | Gyration Radius (m) | 4.5×10^-3^ | | | | | |
| $n$ | Rotating Speed (rpm) | 300 | | | | | |
| $L$ | Normal Forces (N) | 45$\sqrt{2}$ | | | | | |
| *t* | Test Time (s) | 300 | | | | | |
| $k$ | Scale Factor | 0.1 | | | | | |

Table S1. Parameters of the full COMSOL model used in the simulation

| Symbol | Description | Expression |
| --- | --- | --- |
| $v$ | Relative velocity at the contact interface (m/s) | $2\cdot\pi\cdot R\cdot n/60$ |
| $r$ | Contact radius (m) | $r_{hertz}+b\cdot\ln\left(n\cdot t\right)$ |
| *q* | Frictional power per unit contact area (W/m^2^) | $cof\left(t\right)\cdot L\cdot v/\left(k\cdot\pi\cdot r^{2}\right)$ |

Table S2. Variable expression used in the simulation
